# Reduction of Acoustic Reflex Threshold in Neonates without Auditory Risk

**DOI:** 10.1016/S1808-8694(15)30034-3

**Published:** 2015-10-19

**Authors:** Jordana Costa Soares, Renata M.M. Carvallo

**Affiliations:** a Holder of a FAPESP scholarship – Speech and Hearing Therapist; b Associate Professor at the Phonoaudiology program of the FMUSP USP Medical School

**Keywords:** acoustic reflex, newborns, auditory sensitization, facilitating stimulus

## Abstract

The auditory sensitization, a tool used in the investigation of acoustic reflex, allows the decrease of acoustic reflex thresholds from a facilitating stimulus. It may be presented before or simultaneously with the elicitor tone. The thresholds after and before the facilitating stimulus are compared and it is expected to see the decreased threshold. From the study of the acoustic reflex it is possible to obtain information about the auditory pathways, such as structures of the brainstem, since the acoustic reflex pathway is related to the auditory nuclei in this site. They are also involved in auditory processing. Thus, alterations of the acoustic reflex could be related to deficits in auditory processing skills.

**Aim:**

This study aims at investigating the acoustic reflex sensitization from a high-frequency facilitating tone (6 kHz) in newborns without risk factors to hearing impairment.

**Results:**

The acoustic reflex threshold decreased in males and females for all studied frequencies.

**Conclusion:**

A high-frequency facilitating tone presented simultaneously produced a decrease of the acoustic reflex threshold in newborns without risk factors to hearing impairment.

## INTRODUCTION

The acoustic reflex involves the auditory nuclei in the brain stem related to auditory processing (Colletti et al., 1992^1^; Carvallo, 1996^2^). Therefore, acoustic reflex changes may suggest alterations on some auditory nuclei and impair skills related to hearing stimuli processing, such as location, selective attention, speech recognition amidst noise, and frequency selectivity (Carvallo, Albernaz, 1997)^3^.

It is possible to assess the acoustic reflex thresholds of neonates and infants. Vincent, Gerber (1987)^4^ found contralateral acoustic reflex on 92.5% of neonates with up to 48 hours of age and on 95% of infants with up to 6 weeks of age. McMillan et al. (1985)^5^ were also able to identify ipsilateral acoustic reflexes on 85-95% of the ears of 46 infants with ages ranging between 2 weeks and 12 months, all free from hearing alterations. Carvallo, Albernaz (1997)^3^ identified ipsilateral acoustic reflex on 100% of infants included in their cases.

It is possible that there might be indirect multi-synaptic pathways involving other areas of the central nervous system aside from the acoustic reflex arc direct pathways. Borg (1973)^6^ presented evidence of a pathway involving the reticular formation. The role of such pathway is yet unknown, but it may be related to complex characteristics of the acoustic reflex such as delivering anticipatory response, that is, to improve the acoustic reflex response (reducing the threshold) as observed in auditory sensitization procedures.

Acoustic reflex analysis -- as a possibility of investigating the efferent pathway -- depends on the integrity of the auditory afferent and efferent pathways. The auditory sensitization procedure has been used as a tool for acoustic reflex analysis, as it allows the acoustic reflex threshold reduction as of the introduction of a facilitating stimulus. Such stimulus may be introduced before or simultaneously to the tone used to elicit the acoustic reflex. Thresholds before and after exposure to the facilitating tone are compared. The one after exposure is expected to be lower, as reported by various authors.

**Figure 1:**
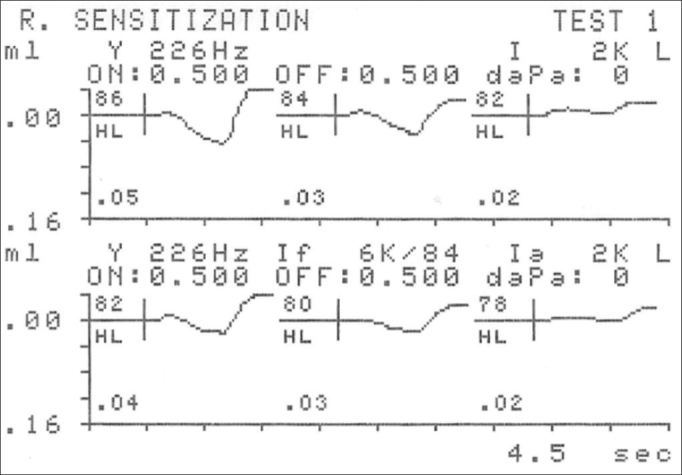
Example of the effect a 6 kHz facilitating tone has on reducing the acoustic reflex threshold.

Figure 1 (below) depicts one example of acoustic reflex sensitization. Initially, the reflex threshold at 2kHz was obtained at 84 dB. When the threshold was assessed again, now with the introduction of a facilitating 6 kHz tone in the intensity at which the acoustic reflex had been identified (84 dB), the threshold drops to 80 dB.

Deutsch (1973)^7^ studied auditory sensitization in adult patients with normal hearing and found reduced acoustic reflex thresholds in most subjects after stimulation by white noise. The author makes reference to the theory that considers the potential of enhancing VIII cranial nerve potential sensitivity by means of stimulation, and mentions the possibility of stimulation leading to fatigue and consequent recruitment (reduction on the acoustic reflex threshold). Sesterhen, Breuninger (1976)^8^ also found reduced acoustic reflex thresholds (30 dB) after the introduction of an 8 kHz tone concurrently to the second assessment of the acoustic reflex thresholds on 25 adult ears. On a later study (1977)^9^, the same authors found reduced acoustic reflex thresholds (20-30 dB) in normal ears and in ears with hearing loss after introducing concurrent 6 or 8 kHz facilitating tones of the same intensity as that of the tone used to elicit the acoustic reflex. Blood, Greenberg (1981)^10^ also identified reduced acoustic reflex thresholds when simultaneously introducing a facilitating tone in a single ear and in both ears.

Stelmachowicz, Gorga (1983)^11^ concluded that the acoustic reflex threshold reduction does not depend on the frequency of the facilitating stimulus. They were able to observe threshold reductions at various frequencies (500 Hz, 1, 2, 4 and 6 kHz), suggesting that the cochlea tonotopic pattern does not interfere in the process. The detected independence from frequency indicates that acoustic reflex facilitation is not primarily mediated by afferent mechanisms. The authors propose that facilitation takes place at the efferent portion of the acoustic reflex arc.

Jeck et al. (1983)^12^ have also observed reduced acoustic reflex thresholds of 10 to 12 dB in adults for frequencies of 500Hz, 1 and 2 kHz using a 6 kHz simultaneous facilitating tone. The authors suggested that sensitization may improve the signal-noise ratio in complex hearing scenarios by attenuating the lower frequencies.

Differently from other authors, Kumar and Barman (2002)^13^ used broad band noise to find increased acoustic reflex thresholds. They introduced contralateral white noise simultaneously to the acoustic reflex threshold assessment. Thresholds were increased by 4 dBNA at 1 kHz and by 3.6 dBNA at 2 kHz. Such effect was imputed to the auditory efferent pathway activation by broad band noise.

Carvallo, Soares (2004)^14^ looked into acoustic reflex sensitization in young women without audiologic complaints and with tonal hearing thresholds within normal ranges. The frequencies analyzed were of 500 Hz, 1, 2 and 4 kHz respectively, while the facilitating tone was of 6 kHz, introduced simultaneously and ipsilaterally. Acoustic reflex thresholds decreased in 6.7 to 13 dBNA on right ears and in 14.3 to 17.2 dBNA on left ears. The observed difference between the values found for right and left ears was not significant for any of the frequencies.

Studies analyzing the activation of the auditory efferent pathway have contributed to enhance the understanding of acoustic reflex reduction in adults. However, its occurrence in neonates still remains doubtful, as it has not been described in papers studying such population. Functional studies of the efferent pathway in neonates using other principles such as the analysis of suppressed otoacoustic emissions (Durante, Carvallo; 2002^15^; During; 2004^16^) have shown that the efferent pathway is present at birth. Therefore it is reasonable to assume that acoustic reflex threshold reduction may also be found in neonates.

## OBJECTIVE

This paper aims at investigating acoustic reflex threshold variations resulting from simultaneous ipsilateral application of a 6 kHz facilitating stimulus in full term birth neonates, without indicated risk for hearing loss and with transient-evoked otoacoustic emissions.

## MATERIALS AND METHOD

### Sample

The sample comprised 40 full term babies, at proper weight for their ages, without prenatal, perinatal, or postnatal complication, and without indicated risk for hearing loss. Twenty babies of each gender were chosen for the study. Tests were carried out within the neonatal period.

The neonates participating in the study –- all under Informed Consent signed by their parents (research approved by the Ethics Committee of the USP University Hospital permit # 174/01) – fit the following inclusion criteria:

1) Presence of TOAE on both ears during hearing screening, according to the criteria set by Brass, Kemp (1994)17, and Letourneau et al. (2000)^18^.
2) Ears with normal tympanometric curves, types A or D (double peak for neonates) and presence of acoustic reflex on at least two frequencies, so that the threshold comparison without and with 6 kHz tone stimulation could be done.

### Materials

• “ILO 292 / ECHOPORT PLUS Otodynamics Analyser” – for the acquisition of OAEs using the ‘Quickscreener’ software, indicated for neonatal hearing screening. This software consists of a standard non-linear mode composed of four stimuli, three of which are equal to one another while the remaining one is reversed and three times greater in terms of amplitude. Response analysis time was of 12ms. Stimuli were of the ‘click’ type, had a duration of 80us and intensity of 80 dB at peak NPS equivalent (Otodynamics, 1992)^19^.
• Portable computer with a Pentium III processor, color monitor, with ILO V5 292 Echoport Plus Otodynamics Analyser installed.
• Grason Stadler TympStar Middle Ear Analizer release 2 immittancemeter – microprocessed and equipped with three tone frequencies probe: 226, 678 and 1000 Hz. Tympanometric measures were carried out automatically by the device at 50 decaPascals per second (daPa/s). Results were plotted in a graph and printed out. The analysis of ipsilateral acoustic reflexes was done with stimuli calibrated in dBHL, played on a loudspeaker assigned exclusively to the ipsilateral mode. The signal was digitally multiplexed, thus allowing the probe tone (226 Hz) to be separated from that of the stimulus avoiding overlapping waves and consequent generation of artifact. The equipment used to analyze ipsilateral acoustic reflex delivers a maximum output of 110 dBHL. It was calibrated for São Paulo city altitude, and all measures were taken from the standpoint of electrical installation so as to meet the manufacturer's technical specifications. (Grason-Stadler, 2001)^20^

### Procedures

The procedures were carried out at the audiology department of the University Hospital of the University of São Paulo, in a quiet environment. The prenatal and neonatal health data and family information in regards to hearing and language skills were obtained by means of interviews with the mothers. Additional data on the prenatal and perinatal periods was collected from the hospital admission charts.

In order for procedures to be carried out, the neonates were at all times held by their mothers, preferably while asleep. Hearing screening – from the acquisition of TOAEs – was done using the ‘Quickscreener’ software, a tool developed specifically for that purpose. The probe, with a rubber tip in its tip, was fit to the external acoustic meatus to acquire the emissions in the first tested ear. The test began only after satisfactory conditions for the stabilization of the stimulus were achieved. Immittance measurements were carried out as subjects passed the criteria set for Hearing Screening of both ears.

Immittance assessment was done starting by the right ear for one half of the subjects in the sample and by the left ear for the other half. Immittance screening comprised studies on tympanometric curve and acoustic reflex at 100 dBHL. The frequency of the tone produced by the immittance probe was of 226 Hz. Neonates who did not present acoustic reflex in the screening mode at 100 dBHL were sent to analysis in diagnostic mode.

After immittance screening, subjects underwent analysis of ipsilateral acoustic reflexes through the device special mode, where sensitization would be carried out further. The frequencies of the analyzed thresholds were 1, 2 and 4 KHz, in 2 dB incremental steps. Tone length was 0.5 s, with on time of 0.5 s and off time of 0.25 s. Sensitization analysis was carried out separately for each of the frequencies mentioned previously after the first investigation of acoustic reflex threshold was completed.

The facilitating stimulus used was an ipsilateral 6 kHz 0.5 second long tone (length equal to that of the reflex-eliciting tone). For each frequency, the intensity of the facilitating stimulus was kept equal to that in which the acoustic reflex threshold had been obtained. The search for the new threshold was done in incremental steps of 2 dB, with simultaneous exposure to the facilitating stimulus (6kHz).

One must bear in mind that the population analyzed by this study presents its inherent difficulties to being tested. Subjects were tested while asleep, and all procedures were carried out rapidly, as they could wake up at any moment. Many published papers report reductions in the acoustic reflex threshold by 10-12 dB (Jeck et al., 1983) and by 6-17 dB (Carvallo, Soares, 2004), to name a few. Thus, the second threshold analysis in this study was done at a frequency 10 dB below the first threshold obtained.

## RESULTS

### Tympanometry (screening mode)

In terms of tympanometric curves, for all 40 neonates, their two ears double peak curves (D) were present in 60% of the cases, while type A curves were found in the remaining 40%. From the two proportions equality test, it was found that there is no significant difference between curve types (p>0.05), as shown in Table 1.

**Table 1:** Percentage of tympanometric curves present.

Tympanometric Curve	RE		LE	
	Qty	%	Qty	%
Double Peak	24	60%	25	60%
A	16	40%	16	40%
Total	40	100%	40	100%
p-value	0,074		0,074	

### Acoustic Reflex and Auditory Sensitization Analysis

In regards to facilitating stimulus, the mean values for the acoustic reflex thresholds without (WO.FS) and with facilitating stimulus (W.FS) for males and females on each frequency for their right and left ears can be seen on tables 2 and 3 respectively. There was no significant difference between males and females, as in both Tables the p values were greater than 5%.

**Table 2:** Acoustic reflex thresholds without and with facilitating stimulus – RE.

Right Ear		Mean	STD	Size	p-value
1 KHz	Female	96,43	10,62	14	0,621
- WO.FS.	Male	94,71	8,54	17	
1 KHz	Female	76,71	21,36	14	0,294
- W.FS.	Male	84,50	18,54	16	
2 KHz	Female	93,29	9,40	14	0,951
- WO.FS.	Male	93,06	10,59	17	
2 KHz	Female	72,29	23,94	14	0,196
- W.FS.	Male	81,47	11,77	15	
4 KHz	Female	91,85	7,77	13	0,914
- WO.FS.	Male	92,18	7,18	11	
4 KHz	Female	74,85	21,61	13	0,295
- W.FS.	Male	82,50	12,51	12

**Table 3:** Acoustic reflex thresholds without and with facilitating stimulus - LE.

Left Ear		Mean	STD	Size	p-value
1 KHz	Female	92,67	9,00	15	0,643
- WO.FS.	Male	94,25	9,77	16	
1 KHz	Female	78,63	20,12	16	0,159
- W.FS.	Male	87,13	12,18	16	
2 KHz	Female	92,38	8,89	16	0,600
- WO.FS.	Male	94,13	9,76	16	
2 KHz	Female	75,87	21,15	15	0,155
- W.FS.	Male	85,20	12,78	15	
4 KHz	Female	91,67	8,86	12	0,219
- WO.FS.	Male	95,47	6,82	15	
4 KHz	Female	75,82	19,05	11	0,112
- W.FS.	Male	86,67	11,77	12	

Tables 4 and 5 show the mean values for the difference between thresholds obtained in the two scenarios of the study, i.e., without facilitating stimulus (W.FS) and with facilitating stimulus (W.FS). Although reduction is slightly higher among females in both ears, the difference is not statistically significant (p>0.05).

**Table 4:** Mean difference without and with facilitating stimulus - RE.

Right Ear	1 KHz		2 KHz		4 KHz	
	Female	Male	Female	Male	Female	Male
Mean	19,71	9,88	21,00	10,40	20,08	11,40
Standard Deviation	21,16	13,85	23,05	7,49	16,71	10,11
Size	14	16	14	15	12	10
p-value	0,138		0,103		0,167

**Table 5:** Mean difference without and with facilitating stimulus - LE.

Left Ear	1 KHz		2 KHz		4 KHz	
	Female	Male	Female	Male	Female	Male
Mean	15,07	7,13	15,73	8,40	15,27	8,33
Standard Deviation	16,95	7,27	16,65	7,49	13,84	11,81
Size	15	16	15	15	11	12
p-value	0,097		0,131		0,209	

## DISCUSSION

Tympanometry screening underscored a distribution of results between two different configurations. Without analyzing them separately by gender, 60% of the right ear tympanometric curves presented double peak (type D) configuration, while 40% were type A. Both are deemed normal for the neonatal population. The same was found for left ears. Another paper (Vincent, Gerber, 1987)^4^ studying infants identified double peak tympanometric curves, which did not prevent the obtainment of acoustic reflexes.

It is important to stress that all subjects included in this study – both males and females – presented acoustic reflex thresholds in at least one of their ears when analyzed in the device off of the screening mode. The goal here was to verify sensitization by comparing thresholds without and with the 6 kHz stimulus. That was possible only because all subjects presented acoustic reflexes.

The mean values found for acoustic reflex without facilitating stimulus ranged between 91 and 96 dBHL. Close values were also found by Carvallo, Albernaz (1997)^3^ in their research on ipsilateral acoustic reflex in a population similar to that of this study. They used frequencies of 1 kHz and 2 kHz, and found values between 96.3 dBHL at 1 kHz and 95.2 dBHL at 2 kHz. McMillan et al. (1985)^5^ also studied acoustic reflexes in infants at 500 Hz, 1 and 2 kHz, finding mean threshold values between 80 and 85 dBHL.

The presentation of a facilitating stimuli brought significant reduction to acoustic reflex thresholds at all frequencies, for both ears of subjects from both genders, as seen in the literature^10^, ^12^, ^14^ of adult populations.

The facts observed in this study agree with various other papers^10^, ^11^, ^12^ insofar as frequency independence is concerned, as sensitization occurred in the neonatal population regardless of the frequency utilized. Further studies are required to determine whether or not the same would happen for other facilitating stimuli.

From what has been explained one might say that, apart from the conventional investigation of the stapedian reflex, it is also possible to assess the acoustic reflex threshold reduction (sensitization) in neonates. As authors agree that this occurrence is related to the auditory efferent pathway^11^, it may be used to assess this same pathway in neonates.

Additionally, the investigation on auditory sensitization in populations with dysfunctional efferent pathways and in individuals with altered auditory processing may be an important diagnostic tool. It will increase the certainty around the location in the auditory system where sensitization or acoustic reflex threshold reduction occurs, thus contributing to further scientific investigation on hearing.

## CONCLUSION

Simultaneous exposure to high-frequency tones leads to reduced acoustic reflex thresholds, suggesting the effect the auditory efferent pathway may have, as early as in the neonatal stage, when activated by a simultaneous and high frequency stimulus.
